# Hurried Dinners

**Published:** 1893-04

**Authors:** 


### HURRIED DINNERS.

It is a mistake to eat quickly. Mastication performed in haste must be imperfect even with the best of teeth, and due admixture of the salivary secretion with the food cannot take place. When a crude mass of inadequately crushed muscular fibre, or undivided solid material of any description, is thrown into the stomach, it acts as a mechanical irritant, and set up indigestion. When the practice of eating quickly, and filling the stomach with unprepared food is habitual, the digestive organ is rendered incapable of performing its proper functions. Either a much larger quantity of food than would be necessary under natural conditions is required, or the system suffers from lack of nourishment. The matter may seem a small one, but it is not so. Just as a man may go on for years with defective teeth, imperfectly masticating his food, and wondering why he suffers from indigestion, so a man may habitually live under an infliction of hurried dinners, and endure the consequent loss of health, without knowing why he is not well,or how easily the cause of his illness might be remedied.

